# Preload Monitoring of Bolted L-Shaped Lap Joints Using Virtual Time Reversal Method

**DOI:** 10.3390/s18061928

**Published:** 2018-06-13

**Authors:** Fei Du, Chao Xu, Guannan Wu, Jie Zhang

**Affiliations:** 1 School of Astronautics, Northwestern Polytechnical University, Xi’an 710072, China; dufei@nwpu.edu.cn (F.D.); guannan_wu@163.com (G.W.); 2 Mechanical Engineering Department, University of Maryland Baltimore County, Baltimore, MD 21250, USA; 3 Department of Mechanical Engineering, University of Bristol, Bristol BS81TR, UK; j.zhang@bristol.ac.uk

**Keywords:** bolt preload monitoring, bolt joints, time reversal, structural health monitoring, guided wave

## Abstract

L-shaped bolt lap joints are commonly used in aerospace and civil structures. However, bolt joints are frequently subjected to loosening, and this has a significant effect on the safety and reliability of these structures. Therefore, bolt preload monitoring is very important, especially at the early stage of loosening. In this paper, a virtual time reversal guided wave method is presented to monitor preload of bolted L-shaped lap joints accurately and simply. In this method, a referenced reemitting signal (RRS) is extracted from the bolted structure in fully tightened condition. Then the RRS is utilized as the excitation signal for the bolted structure in loosening states, and the normalized peak amplitude of refocused wave packet is used as the tightness index (TI_A_). The proposed method is experimentally validated by L-shaped bolt joints with single and multiple bolts. Moreover, the selections of guided wave frequency and tightness index are also discussed. The results demonstrate that the relationship between TI_A_ and bolt preload is linear. The detection sensitivity is improved significantly compared with time reversal (TR) method, particularly when bolt loosening is at its embryo stage. The results also show that TR method is an effective method for detection of the number of loosening bolts.

## 1. Introduction

Bolted joints are widely used in engineering structures such as aerospace and civil structures because of their ease of assembly and high load carrying capacity. However, bolts are frequently subjected to loosening due to inappropriate preloads during installation, time varying external loads during service, or other environment factors [1]. Bolts loosening may lead to the failure of entire structure. Therefore, it is critical to monitor bolt preload at an early stage to ensure the safety and reliability of structures [2]. Structural heath monitoring (SHM) techniques enable effective monitoring of bolt preload [3]. Impedance and guided wave-based damage detection techniques have been widely used for SHM [4]. Electromechanical impedance (EMI) is sensitive to minor changes in the bolt preload. Perera et al. [5] developed a flexible wireless smart sensor framework based on the EMI method, and the system was successfully used for loosening bolt detection. However, the detection area of EMI is limited to the near field of the piezoelectric active sensor [6]. Therefore, guided wave-based SHM techniques have received much interest for bolt preload monitoring, due to their sensitivity to small structural damages and large sensing range [7]. In addition, integrated impedance and guided wave method was also developed by utilizing impedance and guided wave signals simultaneously to enhance the performance of damage diagnosis [4]. 

Because ultrasonic wave transmission is strongly tied to contact status of bolted interface, wave energy dissipation (WED) related methods have been widely studied. To detect fastener integrity in thermal protection panels in space vehicles, Yang and Chang [8] used attenuation energy and speed of guided wave transmitted across jointed interface to assess bolt torque levels and locations of loosening bolt. Their theoretical analysis based on Hertz contact theory and sinusoidal wavy surface model shows that the energy of transmitted guided wave is proportional to the true contact area of jointed interface which mainly depends on bolt preload. Subsequently, Wang et al. [9] used the wave energy propagated across bolted lap joint to monitor bolt preload. However, the energy did not change with bolt torque when the applied torque reaches a certain value, and this is referred to as saturation phenomenon. Similarly, Amerini and Meo [10] calculated the energy of the transmitted guided wave in frequency domain to assess the tightening state of a bolt lap joint. Yang et al. [11] extended the WED method to composite bolted joints. With a scanning laser ultrasound system, Haynes et al. [12] acquired the full-field wave data and calculated the wave energy before and after the lap joint for monitoring bolt torque levels. Unfortunately, saturation phenomena were also observed in all the above experimental studies. On the other hand, due to multi-mode, dispersion, and boundary-reflection of guided waves [13], the response signal at a joint structure is quite complex [10]. Hence, Kędra et al. [14] investigated the effects of excitation frequency, time range of received signal, sensor positions on the preload detection accuracy of the WED method. They pointed out that these parameters must be carefully selected. 

Apart from energy or amplitude of the guided wave, phase shift has also been used for quantifying bolt torques. Zagrai, et al. [15] estimated bolt torques by measuring delays of guided wave transmitted across bolt joint. Their experimental results demonstrated that bolt torque is proportional to phase shift of the guided waves. Subsequently, Doyle et al. [16,17] further studied phase shift of guided wave propagating in a complex panel using piezoelectric sensors sparsely distributed. The results show that the time at which phase shift occurs is related to the distance between the location of loosening bolt and primary wave propagation path. However, changes of the phase shift induced by a bolted joint are rather small and require sensitive equipment with advanced signal processing capabilities [16]. In addition, because received guided waves are very complex, it is difficult to select correct time window and the corresponding wave speed to calculate phase shift and the distance between wave path and damage.

Since guided wave signals are always very complex, Fink et al. [18] extended time reversal method to guided wave monitoring technique. In time reversal approach, a received signal is reversed and reemitted as an excitation signal, then a reconstruction of the input signal can be obtained at the source position. Hence, time reversal method can effectively reduce the influences of dispersion and multi-modal of guided wave. Watkins and Jha [19] adjusted the above time reversal steps, the new procedure requires one transducer to actuate signals and the other transducer acts as a sensor for all signal paths. This method yields identical signals as the original time reversal method and is more convenient. As the time reversal procedure is still complicated, Cai et al. [20] presented a virtual time reversal method and the time reversal procedure was replaced by signal operations. The transfer function in the virtual time reversal method was obtained by taking the derivative of the step pulse responses of a structure. Time reversal-based guided wave monitoring techniques have been widely applied to damage detection for metallic plates [21,22] and composite plates [19,23,24]. Recently, Parvasi et al. [25] proposed to use time reversal (TR) method to focus guided wave energy transmitted through bolt joint, and then used the refocused amplitude peak as tightness index for preload detection. The authors also simulated the proposed detection method by a finite element model considering roughness of contact surfaces. Both numerical and experimental results show that the proposed tightness index increase with bolt torque. However, when the bolt torque is relatively high, the focused signal peak amplitude changes very slightly. Meanwhile, Wang et al. [26] experimentally investigated the TR guided wave method for bolted preload detection. The results demonstrate that as the surface roughness of the bolted interface increases, the saturation phenomenon becomes insignificant. 

The applications of the above bolt preload detection methods were limited to flat bolt lap joint. However, L-shaped bolt joints are more common in real structures [27,28]. As the structure of L-shaped bolt joints are more complicated, complex signal processing methods are always needed. Jalalpour et al. [27] proposed a preload monitoring method for a single 90° bolted joint. Fast Fourier transform, cross-correlation and fuzzy pattern recognition were used to process transmitted waves. However, the fuzzy sets of torque level are limited, and the signal processing procedure is complicated. Montoya et al. [29] assessed rigidity of L-shaped bolt joint using transmitted wave energy. Then, Montoya et al. [30] further extended the method to bolt loosening and preload monitoring of satellite panels jointed by right angle bracket. Their experimental results display that some measurement parameters, such as the time window of received signal, have a significant effect on sensitivity and repeatability of the measurement [30]. In addition, the measurement method is still not very sensitive at the early stage of bolt loosening.

From the above literature review, it can be found that many research efforts have dedicated to bolt preload monitoring. However, the detection sensitivity of guided wave-based techniques is still not good, especially at the early stage of bolt loosening. On the other hand, the current studies mainly focused on flat jointed structures with single bolt, complex bolt jointed structures such as L-shaped bolt lap joints with multiple bolts have not been fully studied. Motivated by Ref. [20], a simple virtual time reversal (VTR) method is developed for accurate bolt preload monitoring in this paper. In this method, a referenced reemitting signal (RRS) is extracted from the bolted structure in fully tightened condition (healthy state). Then the RRS is utilized as the excitation signal for the bolted structure in loosening states (unhealthy states), and the normalized peak amplitude of the received focus wave packet is used as the tightness index (TI_A_). The proposed method is experimentally validated by L-shaped bolt joints with single and multiple bolts and is compared with TR guided wave method. This paper is organized as follows. Section 2 presents the proposed VTR and its theoretical backgrounds. Then, the experiment setup and steps are shown in Section 3. Section 4 compares the preload detection results of the proposed VTR method and TR method. The selections of measurement frequency and tightness index are discussed in Section 5. Finally, conclusions are summarized in Section 6.

## 2. A Virtual Time Reversal Method

### 2.1. The Time Reversal Guided Wave Method

**Figure 1:**
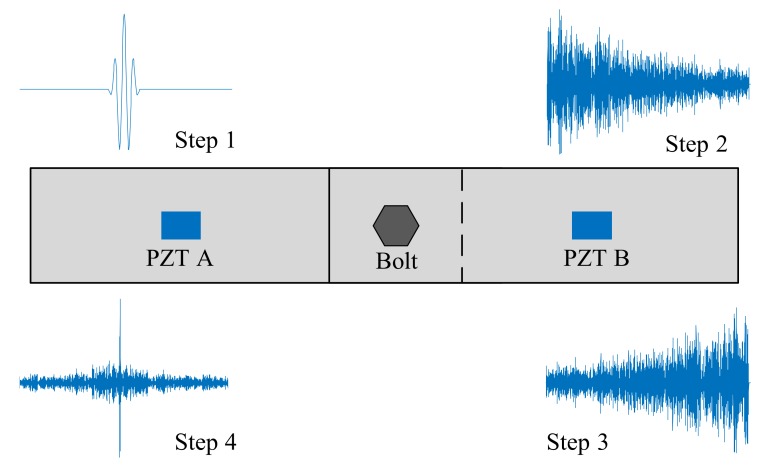
The procedure of the preload monitoring of a bolt lap joint using TR method.

The original time reversal (TR) guided wave method has been used for bolt preload detection. The TR procedure for bolt jointed plates can be divided into four steps, which is shown in Figure 1. Step 1, a tone burst input *e*(*t*) is applied to transducer A, which activates wave propagation in the plate; Step 2, a wave response signal *u*(*t*) is captured by transducer B; Step 3, the recorded signal *u*(*t*) is reversed in time domain and is reemitted using transducer B; Step 4, a guided wave signal is captured by transducer A again, and the original signal is reconstructed [31]. Since the peak amplitude of the reconstructed wave packet can be used for the preload estimation [25]. Therefore, a tightness index (TI_A_) is defined using the following equation:

(1) $${\mathrm{TI}}_{A}=\frac{v_{up}}{v_{hp}}$$

where *v_up_* is the peak amplitude of the reconstructed signal obtained from an unhealthy state, *v_hp_* is the peak amplitude of the reconstructed signal obtained from the healthy state. In this way, the reconstructed signal peaks can be normalized for preload estimation.

Usually, PZT transducers are used for both stimulating and capture guided waves. Therefore, while a tone pulse input is emitted from PZT A, the response signal at the sensor PZT B can be expressed as the following equation [23]:

(2) $$V_{B}\left(\omega \right)=V_{A}\left(\omega \right)G\left(\omega \right)$$

where *V_A_*(*ω*), *V_B_*(*ω*), and *G*(*ω*) are the input signal at PZT A, received signal at PZT B and structure transfer function for the given wave propagation path. In step 3, the time reversed signal can be written as

(3) $$V_{B}^{*}\left(\omega \right)=V_{A}^{*}\left(\omega \right)G^{*}\left(\omega \right)$$

where the superscript * denotes the complex conjugate operation. The transfer function from PZT A to PZT B is assumed to be the same from B to A [31]. Hence, after this time reversal signal is reemitted from PZT B to A, the reconstructed signal received at PZT A is

(4) $$V_{rc}\left(\omega \right)=V_{A}^{*}\left(\omega \right)G^{*}\left(\omega \right)G\left(\omega \right)$$

where G^*^(*ω*)*G*(*ω*) is refer to as time reversal operator. Therefore, the phase-delay factor in the time reversal operator is removed. For a single mode wave, the transducer function can be expressed as [32]

(5) $$G\left(\omega \right)=A\left(\omega \right)e^{-ik\left(\omega \right)r}$$

where *i*, *k*(*ω*) and *r* are imaginary unite, wavenumber of the mode and wave propagation distance between A and B. Hence, Equation (4) can be written as

(6) $$V_{rc}\left(\omega \right)=V_{A}^{*}\left(\omega \right){\left|A\left(\omega \right)\right|}^{2}$$

It can be found that the shape of the reconstructed signal is the same as that of the time reversed original signal when the transfer function is independent of frequency. Transfer function is frequency dependent [33], so a narrowband input signal is usually used to enhance time reversal progress [23].

The transfer function *G*(*ω*) is corresponding to the impulse response function in time domain. For a bolt jointed structure shown in Figure 1, the transmitted wave energy between the two PZT sensors is proportional to the true contact area of the interface [8]. Hence, as the bolt preload increase, the energy of the impulse response increases and the amplitude of the corresponding *G*(*ω*) increases. Accordingly, the reconstructed signal peak obtained by the time reversal method increase at the same time. Thereby, time reversal method can be used for bolt preload monitoring [25,26]. However, due to the saturation of true contact area at high bolt preload [25,26], the TR method lost its detection sensitivity, especially at relative high bolt preload cases.

### 2.2. A Virtual Time Reversal Method for Bolt Preload Monitoring

**Figure 2:**
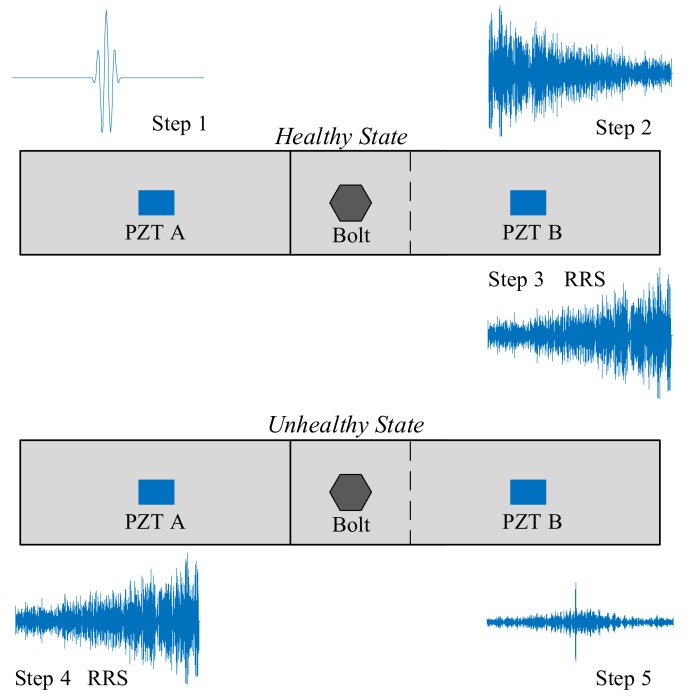
The procedure of the preload monitoring of a bolt lap joint using the VTR method.

To improve the bolt preload detection sensitivity, a virtual time reversal (VTR) method is proposed. In this method, a referenced reemitting signal (RRS) is obtained from the bolted structure in healthy state. The procedure of this method is shown in Figure 2. Step 1, a bolted structure in healthy state is used, and a tone burst signal is applied to transducer A on this structure; Step 2, a response signal is captured by transducer B; Step 3, the response signal is reversed in time domain and is recorded as a RRS of this bolted jointed structure; Step 4, for the same structure in unhealthy states (unknown preloads), the RRS is stimulate using transducer A; Step 5, the final response signal is captured by transducer B. If the bolt is not loosened, the final received signal will be refocused at PZT B and will be similar to the original tone burst signal. Otherwise, the final received signal will not be refocused, and the peak amplitude will change significantly. Therefore, the peak amplitude of this received signal can be used for preload detection. In this way, the complicated TR procedure is simplified. Moreover, the RRS is also emitted in the healthy state to get a reconstructed wave packet to normalize all the peak amplitudes measured under other torques. Hence, the corresponding tightness index TIA can also be calculated by Equation (1).

Like Equation (3), the RRS in the VTR method can be expressed as

(7) $$R\left(\omega \right)=V_{A}^{*}\left(\omega \right)G_{h}^{*}\left(\omega \right)$$

where *G_h_*(*ω*) is the transfer function of the healthy state for the given path. The transfer function changes due to the change of bolt preload. Hence, the final received signal in step 5 is

(8) $$V_{rm}\left(\omega \right)=V_{A}^{*}\left(\omega \right)G_{h}^{*}\left(\omega \right)G_{uh}\left(\omega \right)$$

where *G_uh_*(*ω*) is the transfer function of the unhealthy state, and G^*^_h_(*ω*)*G_uh_*(*ω*) is time reversal operator in VTR.

Compared with the *G_h_*(*ω*), not only the amplitude of *G_uh_*(*ω*) becomes smaller, the phase of *G_uh_*(*ω*) also changes due to the change of the bolt preload. Hence, the phase-delay factor in the time reversal operator is not completely removed. For this VTR method, the bolt preload not only affects the amplitude of wave packets, but also the refocusing effectiveness. Consequently, the measurement sensitivity can be improved. 

## 3. Experimental Setup and Procedure

### 3.1. Experimental Setup and Specimens

**Figure 3:**
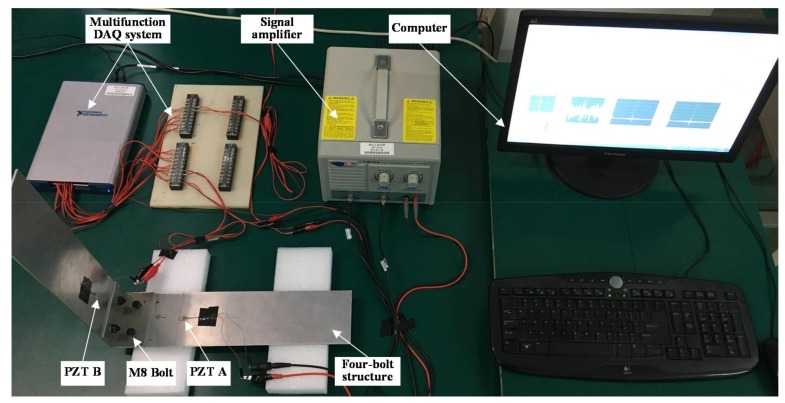
Experimental setup.

Experiments were carried out to investigate the effectiveness of the proposed VTR method and to compare with TR method. The experiment apparatus and specimens are shown in Figure 3. A torque wrench with a resolution of 0.2 Nm was used to apply bolt load. M8 bolts and nuts were used to fasten aluminum plates of type Al-5052. Considering the allowable tensile load of M8 bolt and the yielding strength of the selected aluminum alloy, 16 N·m was selected to be the fully tightened torque. In addition, the joint under hand tight corresponded to a fully loosened condition. A multifunction data acquisition system NI USB-6366 was used to generate and record wave signals. The sampling frequency was 2 MHz. Programs built in LabVIEW environment were used to perform the TR and VTR methods. A high voltage amplifier PINTEK HA-400 was selected to amplify excitation signals. PZT patches were mounted on the top surface of the specimens and were utilized as actuator and sensor, as shown in Figure 3. The type of the PZT patch is PZT-5H. 

**Figure 4:**
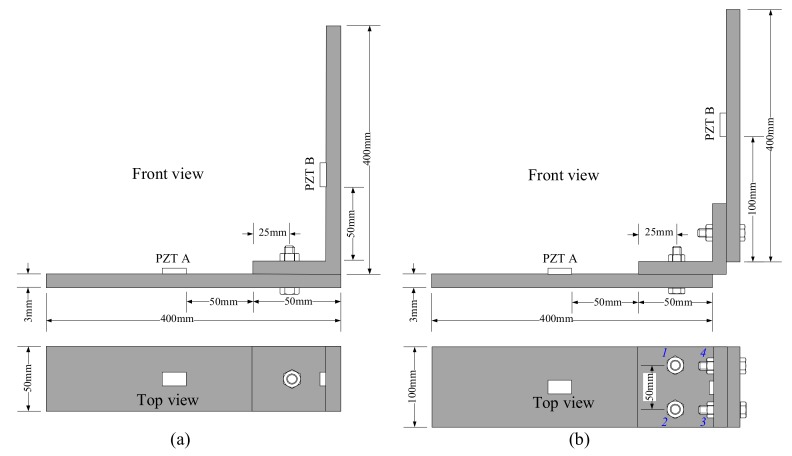
Schematic of experimental specimens (**a**) single bolt structure; (**b**) four-bolt structure.

Two sets of L-shaped bolted structures jointed by one and four bolts were measured, and they are named single bolt structure and four-bolt structure, respectively. The dimensions of the specimens are shown in Figure 4. In Figure 4b, the bolts are numbered from 1 to 4.

### 3.2. Experimental Procedure

For the single bolt structure, 10 bolt torque levels were measured which are from hand tight to 16 Nm. For the four-bolt structure, 6 torque levels of bolt 2 from hand tight to 16 Nm were measured first. At this time, the torques of bolt 1, 3 and 4 were all 16 Nm. Then different numbers of loosening bolts were measured, and the corresponding loosening cases are shown in Table 1. Both the TR and VTR methods were used for every bolt torque measurement for comparison. 

3.5-peak tone burst signals with central frequencies from 100 kHz to 300 kHz were used as original inputs to investigate the effect of frequency. Since the frequencies are relative low, only A_0_ and S_0_ mode were excited in 3 mm thick aluminum plate. The signals were amplified to 40 V_pp_ (peek-to-peek) and sent to PZT A as excitation. In addition, the specimens were placed on a foam support during the experiment, so that the boundary condition can be idealized as free-free. Both bolt structures were dissembled and resembled several times, so that every bolt torque case was measured repeatedly three times. As contact surfaces also affect contact characteristics of the jointed interface [34], the contact surfaces were cleaned by acetone before assembly. For every measurement, each signal was recorded 5 ms length and was averaged 16 times to reduce noise. Please note that temperature-induced propagation speed change has a significant effect on the ultrasonic signal [35]. Therefore, to remove the effect of temperature, the temperature was 20 ± 1 °C during the experiments.

## 4. Results

### 4.1. Results of the Single Bolt Structure

Experimental results of the single bolt structure are presented here. Each torque level was measured using both TR and VTR methods at the same time for comparison. 

#### 4.1.1. The Time Reversal Method

**Figure 5:**
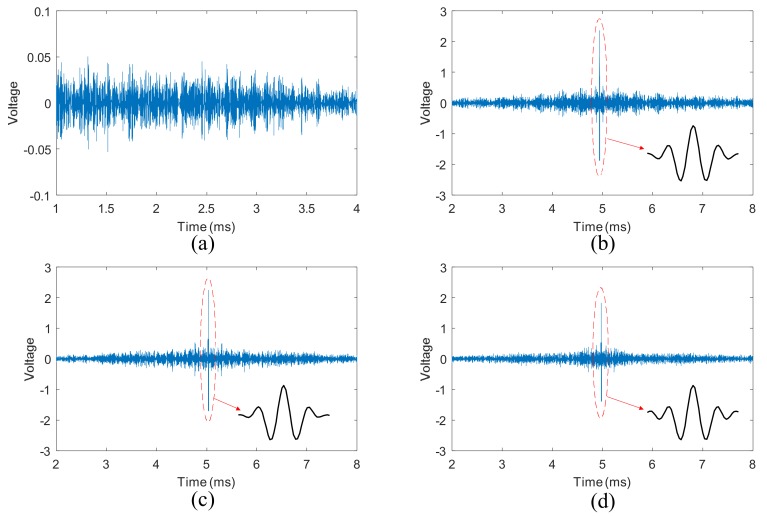
Reconstructed signals using TR method acquired from single bolt structure: (**a**) response signal in step 2; (**b**) reconstructed signal with 14 Nm torque; (**c**) reconstructed signal with 10 Nm torque; (**d**) reconstructed signal with 2 Nm torque.

The response signal at PZT B in step 2 of TR method is shown in Figure 5a, when the central frequency of the excitation tone burst signal is 150 kHz and the bolt torque is 10 Nm. The corresponding reconstructed signal in step 4 is shown in Figure 5c. When the bolt torques are 14 Nm and 1 Nm, the reconstructed signals are shown in Figure 5b,d, respectively. The shape the refocused wave packets are shown in Figure 5b–d.

From Figure 5a, it can be seen that the response signal is very complicated. In contrast, the shape of the refocused wave packets is almost the same as the excitation signal, as shown in Figure 5b–d. The input signal can be reconstructed even though the bolt torque is relatively small (2 Nm). Meanwhile, the peak amplitude of the reconstructed signal at 2 Nm is smaller than that at 8 Nm. 

**Figure 6:**
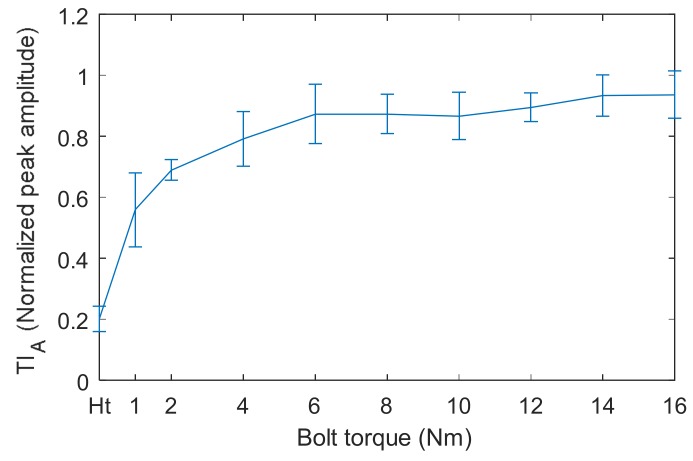
TI_A_ measured by TR method versus bolt torque for single bolt structure.

The tightness index (TIA) are presented in Figure 6. It can be clearly seen that TI_A_ does not change with bolt torque when the applied torque reaches 6 Nm. This is a serious saturation phenomenon, and the bolt torque level cannot be distinguished from each other at this time. The saturation phenomenon in Figure 6 is consistent with previous experimental results [25,26]. On the other hand, there are deviations in the results, although the temperature has been controlled during the experiment. The main reasons might be the inaccuracies in the measuring path and bolt preload. In the repeated experiments, the bolt jointed structures were disassembled and reassembled three times. In this way, the wave propagation path might be changed due to assembly variations [36]. The other main source of deviation is that there is a scatter in the torque-preload relationship [1], and the preloads were not the same when the bolt torques were the same in the repeated experiments. 

#### 4.1.2. The Virtual Time Reversal Method

**Figure 7:**
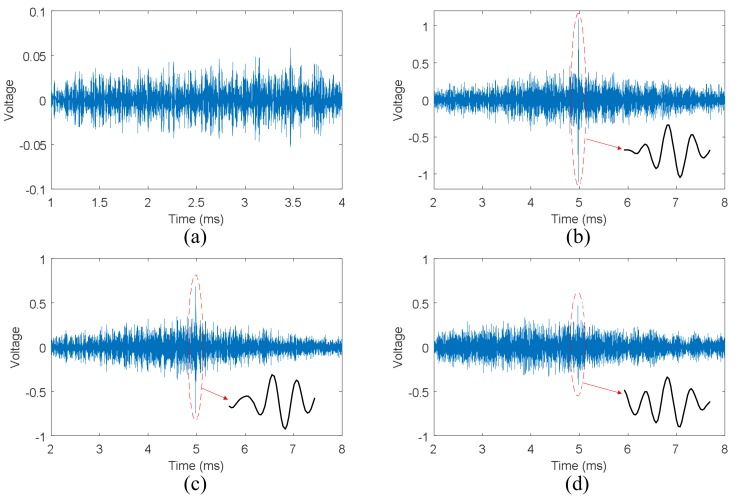
Reconstructed signals using VTR method acquired from single bolt structure: (**a**) RRS signal obtained in the healthy state; (**b**) reconstructed signal with 14 Nm torque; (**c**) reconstructed signal with 10 Nm torque; (**d**) reconstructed signal with 2 Nm torque.

The RRS signal obtained from the healthy state using the VTR method is illustrated in Figure 7a, and the central frequency of the input signal is still 150 kHz. The reconstructed signals in step 5 of VTR method are shown in Figure 7b–d, the corresponding bolt torques are 14 Nm, 10 Nm, and 2 Nm. The shapes of the refocused wave packets are also shown in Figure 7b–d. 

From Figure 7b, it can be clearly seen that the wave is refocused on the original source point A. Compared with Figure 5b,c, the peak amplitude in Figure 7b is much smaller than the corresponding result of TR method shown in Figure 5b. Moreover, the amplitude of the refocused wave packet decreases with bolt torque sharply according to Figure 7b–d. On the other hand, the shapes of refocused wave packets shown in Figure 7b–d are different from those shown in Figure 5b–d. The reason is that besides amplitude, the phase of the transfer function of unhealthy state is different from that of the transfer function of healthy state. In addition, this difference increases with the decrease of bolt torque. Hence, the refocusing capability of VTR method decreases with bolt torque noticeably according to Equation (8). 

**Figure 8:**
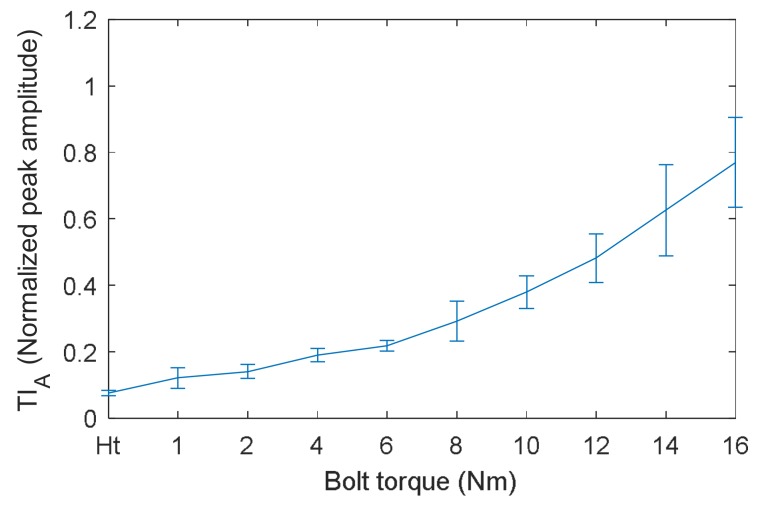
TI_A_ measured by VTR method versus bolt torque.

The TI_A_ obtained by VTR method are displayed in Figure 8. It can be clearly observed that the relationship between TI_A_ and bolt torque is almost linear, and the saturation phenomenon shown in Figure 6 is completely overcame by VTR method. This linear relationship indicates a good measurement sensitivity of VTR method, especially at the early stage of bolt loosening. Hence, it is possible to the distinguish bolt torque levels from each other using VTR method. 

### 4.2. Results of the Four-Bolt Structure

Experimental results of the four-bolt structure are presented here. First, 6 torque levels of bolt 2 were measured, then different numbers of loosening bolts were measured. Each tight condition was measured using both TR and VTR methods at the same time for comparison. 

#### 4.2.1. The Time Reversal Method

**Figure 9:**
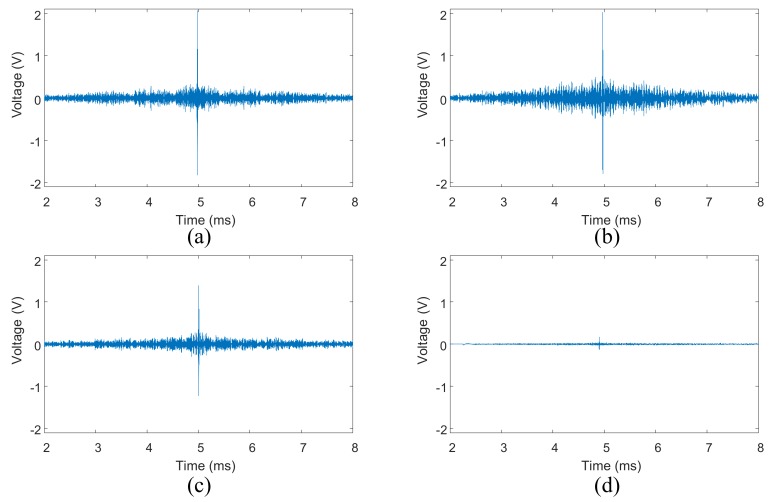
Reconstructed signals using TR method acquired from four-bolt structure: (**a**) reconstructed signal with 8 Nm torque of bolt 2; (**b**) reconstructed signal with 1 Nm torque of bolt 2; (**c**) reconstructed signal under loosening case 5; (**d**) reconstructed signal under loosening case 1.

Four reconstructed signals in step 4 of TR method are shown in Figure 9, the central frequency of the excitation burst signal is 200 kHz. The corresponding tight conditions are 8 Nm torque of bolt 2, 1 Nm torque of bolt 2, loosening case 5 and case 1 listed in Table 1. 

From Figure 9a,b, it can be seen that the peak amplitude of the reconstructed signal decreases little with torque of single bolt. However, the peak amplitude decreases obviously when there are two loosening bolts (Case 5), as shown in Figure 9c. It can also be seen that the input signal can still be refocused even though all four bolts are loosening (Case 1). As expected, the peak amplitude becomes very small at this time. 

**Figure 10:**
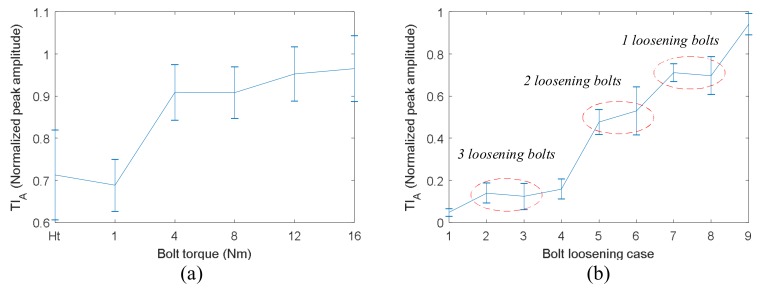
TI_A_ measured by TR method versus bolt tight condition for four-bolt structure: (**a**) different torques of bolt 2; (**b**) different loosening cases.

The results of TI_A_ are presented in Figure 10. Figure 10a shows the TI_A_ versus torque of bolt 2. It can be clearly seen that bolt torques cannot been distinguished from each other by TI_A_ when the bolt torque is greater than 4 Nm. Figure 10b displays the TI_A_ versus bolt loosening case listed in Table 1. It can be observed that TI_A_ decreases significantly with the increase of loosening bolt number. Although case 4, 5 and 6 mean 2 bolts loosening, the peak amplitude of case 4 is much smaller that these of case 5 and 6. The reason is that the loosening bolts in case 4 (bolt 1 and 2) were located on the same side of the right angle of the bracket, while the loosening bolts in case 5 and 6 were located on both sides of the right angle. This has a significant effect on the energy transfer across the bolt interfaces. Hence, the peak amplitude of case 4 is the same as these of case 2 and 3. Therefore, TR method is an effective method for detection of the number of loosening bolts. However, it cannot be used for monitoring the early stage of bolt loosening when one bolt only loses part of its preload. 

#### 4.2.2. The Virtual Time Reversal Method

**Figure 11:**
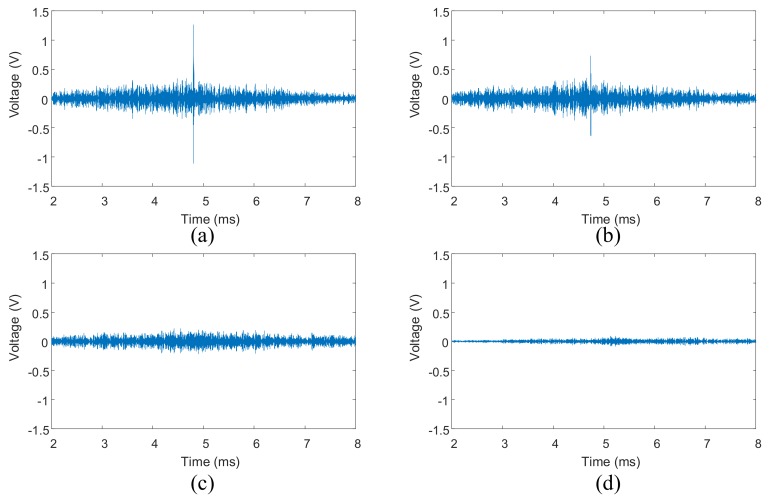
Reconstructed signals using VTR method acquired from four-bolt structure: (**a**) reconstructed signal with 8 Nm torque of bolt 2; (**b**) reconstructed signal with 1 Nm torque of bolt 2; (**c**) reconstructed signal under loosening case 5; (**d**) reconstructed signal under loosening case 1.

Four reconstructed signals in step 5 of VTR method are shown in Figure 11, the central frequency of the input signal is also 200 kHz. The corresponding tight conditions are also 8 Nm of bolt 2, 1 Nm of bolt 2, loosening case 5 and case 1. 

Figure 11a,b display that the ultrasonic waves are still refocused on the original source when the torques of bolt 2 are 8 Nm and 1 Nm. In addition, the peak amplitude of the reconstructed signal decreases significantly with bolt torque. This is different from the results shown in Figure 9a,b. Meanwhile, the peak amplitude in Figure 11b is much smaller than that shown in Figure 9b obtained by TR method, although the bolt torques are the same. From Figure 11c,d, it can be observed that the VTR method almost loses refocusing capability when the loosening bolt number increases to 2. Figure 11c,d also display that the amplitude in the middle segment of the signal is still relative larger than that in other segments, and this maximum amplitude decreases with the increases of loosening bolt number. 

**Figure 12:**
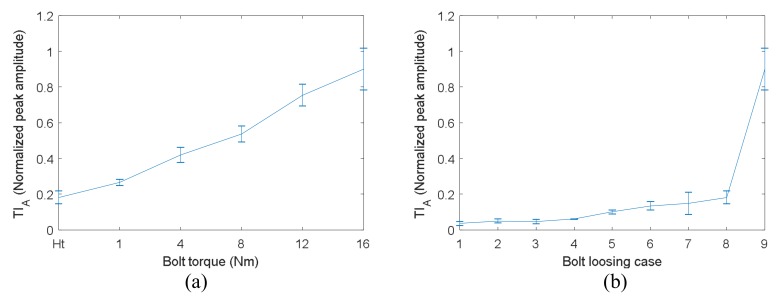
TI_A_ measured by VTR method versus bolt tight condition for four-bolt structure: (**a**) different torques of bolt 2; (**b**) different number of loosening bolts.

The TI_A_ of VTR method were obtained and are displayed in Figure 12. Figure 12a presents the TI_A_ versus torque of bolt 2. It can be clearly seen that the relationship between TI_A_ and bolt torque is linear, and the saturation phenomenon shown in Figure 10a is completely overcame by the proposed VTR method. This linear relationship indicates a high measurement sensitivity of VTR method for bolt preload detection. In addition, the standard deviations of the measurements are relative small, so the bolt torque level can be distinguished from each other easily. In general, the results suggest that the VTR method is accurate enough for the monitoring the early stage of bolt loosening when one bolt only loses part of its preload. Figure 12b displays TI_A_ versus bolt loosening case listed in Table 1. It can be observed that the peak amplitude decreases slowly with the increase of loosening bolt number. The detection sensitivity is relatively low. The reason is that when there are 2 or more loosening bolts, the VTR method almost loses its refocusing capability. 

## 5. Discussion

It is well known that the selections of ultrasound frequency and tightness index are very important for bolt loosening or preload detection. Hence, the effects of frequency and tightness index are discussed in this section.

### 5.1. The Effect of Frequency

**Figure 13:**
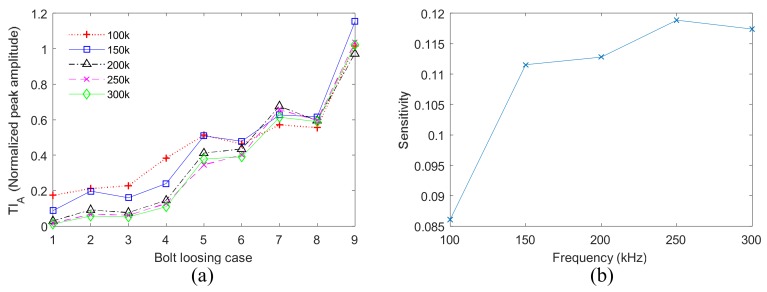
The effect of frequency on TR method (**a**) results using different frequencies; (**b**) detection sensitivity.

The effect of excitation signal frequency on TR and VTR methods is evaluated. Excitation frequencies from 100 kHz to 300 kHz were investigated. As the above results show, the TR method is effective for the detection of bolt loosening number. Therefore, bolt loosening number of four-bolt structure were measured by TR method using different frequencies, and the results are presented in Figure 13a. All the frequencies can be used for loosening number detection. Figure 13a also shows that when the central frequency is 100 kHz, the normalized peak amplitudes obtained under different loosening cases are relatively close. Hence, frequency of 100 kHz is not sensitive for bolt loosening detection compared with other frequencies. Subsequently, the detection sensitivities of all the frequencies are evaluated by the following means. Every curve shown in Figure 13a is fitted by a straight line, and the slope of the line are taken as detection sensitivity of the corresponding frequency. The results are shown in Figure 13b. It can be observed that the detection sensitivity generally increases with ultrasound frequency, especially when the frequencies are from 100 kHz to 250 kHz. 

**Figure 14:**
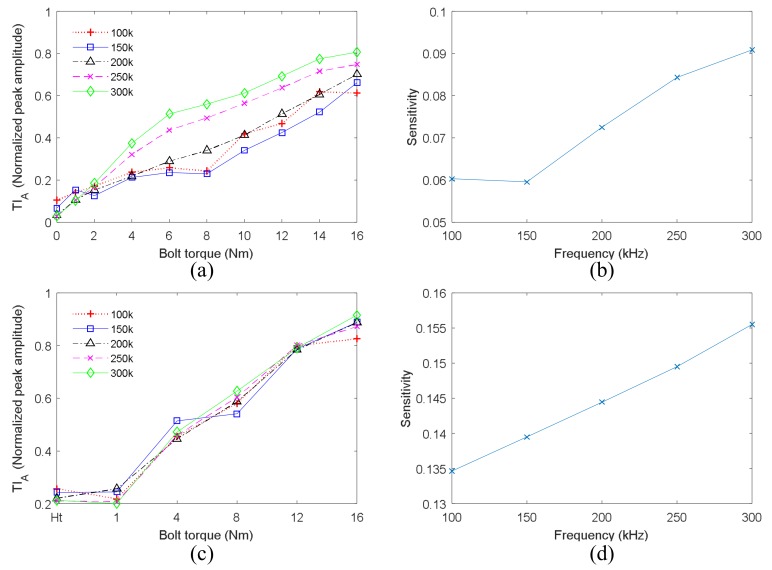
The effect of frequency on VTR method (**a**) results of the single bolt structure; (**b**) detection sensitivity for the single bolt structure; (**c**) results of four-bolt structure; (**d**) detection sensitivity for the four-bolt structure.

The blot preload of the single bolt structure and of bolt 2 in the four-bolt structure were detected by VTR method using different frequencies. The results are shown in Figure 14a,c, respectively. The TI_A_ obtained by all the frequencies increase with bolt preload, and their relationships are generally linear. Hence, frequencies from 100 kHz to 300 kHz can all be utilized for preload detection. In addition, the measurement sensitivities of all frequencies are evaluated using the pervious means, as shown in Figure 14b,d. The sensitivity increases with frequency as well. Therefore, it can be concluded that higher frequency is better for measurement.

The main reason for the trends of sensitivity is that wave energy transmitted through bolt joint is proportional to true contact area of the interface. Meanwhile, true contact area is the summation of micro contact asperities. Since the size of an asperity on a contact surface is relative small (normally smaller than 1 mm), ultrasound wave with smaller wavelength is more sensitive to true contact area [37]. In addition, as the selected frequencies are relatively low, only A_0_ and S_0_ mode are excited in 3 mm thick aluminum plate. When the excitation frequency is from 100 kHz to 300 kHz, wavelengths of A_0_ and S_0_ lamb waves in 3 mm thick aluminum plate decrease significantly with frequency. Hence, the sensitivity of preload measurement increases with guided wave frequency. 

### 5.2. The Selection of Tightness Index

Other than peak amplitude of the reconstructed signal, energy of the transmitted ultrasound signal can also be taken as tightness index. In the discrete time domain, the energy of a received ultrasound signal in time domain [*t_s_, t_f_*] can be simplified as [8]:

(9) $$E=\frac{2\pi }{\omega_{s}}\sum_{t=t_{s}}^{t=t_{f}}V{\left(t\right)}^{2}$$

where *V*(*t*) and *ω_s_* denote the voltage of the discrete sensor signal and its sampling frequency, respectively. In this section, the normalized energy of the refocused wave packet is used as tightness index (TI_E_) to compare with the previous TI_A_. The length of the refocused wave packet was chosen as the corresponding excitation tone bust signal. The energy of wave packets was also normalized by reference measurements of the jointed structure in healthy state to calculate TI_E_. Thus, TI_E_ can be expressed as:

(10) $${\mathrm{TI}}_{E}=\frac{E_{u}}{E_{h}}$$

where, *E_u_* is the energy of the refocused wave packet obtained from an unhealthy state and *E_h_* is the energy of the refocused wave packet obtained from the healthy state.

**Figure 15:**
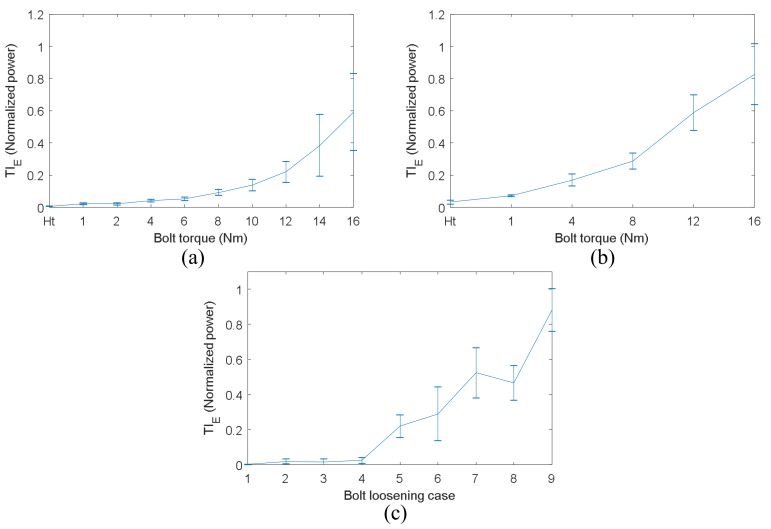
Results of TIE: (**a**) TI_E_ versus bolt torque in the single bolt structure; (**b**) TI_E_ versus torque of bolt 2 in the four-bolt structure; (**c**) TI_E_ versus bolt loosening case in the four-bolt structure.

Figure 15a,b show TI_E_ vs. torques of the bolt in the single bolt structure and of bolt 2 in the four-bolt structure, respectively. Both were measured by VTR method. Figure 15c shows TIE measured by TR method versus bolt loosening number in the four-bolt structure. Please note that the ultrasonic signals used to calculate TI_E_ are the same as those to obtain the results shown in Figure 8, Figure 10b and Figure 12a. 

It can be clearly observed that the linearity of the relationships between TI_E_ and bolt torque shown in Figure 15a,b is not as good as the linearity of those shown in Figure 8 and Figure 12a. From Figure 15c, it can be seen that the TI_E_ of case 1 which is 4 bolts loosening is very close to those of case 2 and 3 which are both 3 bolts loosening. At the same time, the difference between case 7 and 8 is relatively large, although both case 7 and 8 mean 1 bolt loosening. In addition, the standard deviation of TI_E_ is greater than that of TI_A_ when bolt torque is relatively high, as shown in Figure 15. Hence, it can be concluded that the sensitivity and accuracy of TI_E_ are not as good as TI_A_, so TI_A_ is a better selection of tightness index.

## 6. Conclusions

In this paper, a virtual time reversal (VTR) guided wave method is presented to monitor bolt preload accurately, and the complicated TR procedure is simplified. The effectiveness of the proposed method is experimentally validated by L-shaped bolt joints with single and multiple bolts. Based on the results presented in this article, the following conclusions can be drawn:

(1) The relationship between bolt torque and the peak amplitude of refocused wave packet measured by the VTR method is almost linear. Preload detection sensitivity is improved significantly compared with the TR method, especially at early stage of bolt loosening. The saturation phenomenon is completely overcome by the VTR method. The main reason is that both the amplitude and phase of time reversal operator in VTR change with bolt preload. Hence, the bolt preload not only affects the amplitude of wave packets, but also the refocusing capability.
(2) For the VTR method, the original excitation tone burst signal can be refocused at the original source point when the bolt torque is close to healthy state. Nevertheless, the shape of the refocused signal is different from the tone burst signal. In addition, the VTR method almost loses refocusing capability when the loosening bolt number increases to 2. The results also show that the TR method is an effective method for detection of the number of loosening bolts.
(3) The selections of guided wave frequency and tightness index are discussed. Detection sensitivity of VTR and TR methods generally increases with ultrasound frequency. The main reason is that ultrasound wave with smaller wavelength is more sensitive to true contact area, and the wavelength of the selected lamb wave decreases significantly with frequency. In addition, both wave energy and peak amplitude of refocused wave packet were used as tightness index. The sensitivity and accuracy of peak amplitude is better.

PZT sensors can be used for guided waves and EMI monitoring and the two techniques have their own technical merits. The combination of the two techniques is effective to improve the performance of damage monitoring [4,38]. Therefore, how to combine the proposed guided wave method with EMI is the future research work that will enhance the performance of bolt preload monitoring.

## Figures and Tables

**Table 1:** Bolt loosening cases for the four-bolt structure.

Case Number	1	2	3	4	5	6	7	8	9
Hand tight bolt	All	2,3,4	1,2,3	1,2	2,3	1,3	4	2	None

## References

[1] Zhu L., Hong J., Jiang X. (2016). On controlling preload and estimating anti-loosening performance in threaded fasteners based on accurate contact modeling. Tribol. Int..

[2] He K., Zhu W.D. (2014). Detecting Loosening of Bolted Connections in a Pipeline Using Changes in Natural Frequencies. J. Vib. Acoust..

[3] Giurgiutiu V., Lin B., Santoni-Bottai G., Cuc A. (2011). Space application of piezoelectric wafer active sensors for structural health monitoring. J. Intell. Mater. Syst. Struct..

[4] An Y.K., Sohn H. (2012). Integrated impedance and guided wave based damage detection. Mech. Syst. Signal Process..

[5] Perera R., Pérez A., García-Diéguez M., Zapico-Valle J.L. (2017). Active Wireless System for Structural Health Monitoring Applications. Sensors.

[6] Zagrai A., Doyle D., Gigineishvili V. (2010). Piezoelectric wafer active sensor structural health monitoring of space structures. J. Intell. Mater. Syst. Struct..

[7] Lowe P.S., Duan W., Kanfoud J., Gan T.H. (2017). Structural Health Monitoring of Above-Ground Storage Tank Floors by Ultrasonic Guided Wave Excitation on the Tank Wall. Sensors.

[8] Yang J., Chang F.-K. (2006). Detection of bolt loosening in C–C composite thermal protection panels: I. Diagnostic principle. Smart Mater. Struct..

[9] Wang T., Song G., Wang Z., Li Y. (2013). Proof-of-concept study of monitoring bolt connection status using a piezoelectric based active sensing method. Smart Mater. Struct..

[10] Amerini F., Meo M. (2011). Structural health monitoring of bolted joints using linear and nonlinear acoustic/ultrasound methods. Struct. Health Monit..

[11] Yang B., Xuan F.Z., Xiang Y., Li D., Zhu W., Tang X., Xu J., Yang K., Luo C. (2017). Lamb Wave-Based Structural Health Monitoring on Composite Bolted Joints under Tensile Load. Materials.

[12] Haynes C., Yeager M., Todd M., Lee J.-R. (2014). Health Monitoring of Structural and Biological Systems 2014. Monitoring Bolt Torque Levels through Signal Processing of Full-Field Ultrasonic Data.

[13] Dworakowski Z., Ambrozinski L., Stepinski T. (2016). Multi-stage temperature compensation method for Lamb wave measurements. J. Sound Vib..

[14] Kedra R., Rucka M. (2015). Research on assessment of bolted joint state using elastic wave propagation. J. Phys. Conf. Ser..

[15] Zagrai A., Doyle D., Arritt B. (2008). Embedded nonlinear ultrasonics for structural health monitoring of satellite joints. Proc. SPIE.

[16] Doyle D., Zagrai A., Arritt B., Çakan H. (2010). Damage detection in bolted space structures. J. Intell. Mater. Syst. Struct..

[17] Doyle D., Zagrai A., Arritt B. Bolted joint integrity for structural health monitoring of responsive space satellites. Proceedings of the 50th AIAA SDM Conference.

[18] Ing R.K., Fink M. (1998). Time-reversed Lamb waves. IEEE Transactions on Ultrasonics, Ferroelectrics, and Frequency Control.

[19] Watkins R., Jha R. (2012). A modified time reversal method for Lamb wave based diagnostics of composite structures. Mech. Syst. Signal Process..

[20] Cai J., Shi L., Yuan S., Shao Z. (2011). High spatial resolution imaging for structural health monitoring based on virtual time reversal. Smart Mater. Struct..

[21] Poddar B., Kumar A., Mitra M., Mujumdar P.M. (2011). Time reversibility of a Lamb wave for damage detection in a metallic plate. Smart Mater. Struct..

[22] Zeng L., Lin J., Huang L., Zhao M. (2016). Amplitude Dispersion Compensation for Damage Detection Using Ultrasonic Guided Waves. Sensors.

[23] Park H.W., Sohn H., Law K.H., Farrar C.R. (2007). Time reversal active sensing for health monitoring of a composite plate. J. Sound Vib..

[24] Zeng L., Lin J., Huang L. (2017). A Modified Lamb Wave Time-Reversal Method for Health Monitoring of Composite Structures. Sensors.

[25] Parvasi S.M., Ho S.C.M., Kong Q., Mousavi R., Song G. (2016). Real time bolt preload monitoring using piezoceramic transducers and time reversal technique—A numerical study with experimental verification. Smart Mater. Struct..

[26] Wang T., Liu S., Shao J., Li Y. (2016). Health monitoring of bolted joints using the time reversal method and piezoelectric transducers. Smart Mater. Struct..

[27] Jalalpour M., El-Osery A.I., Austin E.M., Reda Taha M.M. (2014). Health monitoring of 90° bolted joints using fuzzy pattern recognition of ultrasonic signals. Smart Mater. Struct..

[28] He K., Zhu W.D. (2011). Finite Element Modeling of Structures with L-Shaped Beams and Bolted Joints. J. Vib. Acoust..

[29] Montoya A.C., Maji A.K. (2011). An Assessment of Joint Rigidity with Ultrasonic Wave Energy. J. Nondestruct. Eval..

[30] Montoya A., Doyle D., Maji A., Dumm H.-P. (2014). Ultrasonic Evaluation of Bolted Connections in Satellites. Res. Nondestruct. Eval..

[31] Park H.W., Kim S.B., Sohn H. (2009). Understanding a time reversal process in Lamb wave propagation. Wave Motion.

[32] Xu B., Giurgiutiu V. (2007). Single Mode Tuning Effects on Lamb Wave Time Reversal with Piezoelectric Wafer Active Sensors for Structural Health Monitoring. J. Nondestruct. Eval..

[33] Zeng L., Lin J., Bao J., Joseph R.P., Huang L. (2017). Spatial resolution improvement for Lamb wave-based damage detection using frequency dependency compensation. J. Sound Vib..

[34] Lin Q., Li B. (2015). Comparison of the influences of surface texture and boundary slip on tribological performances. Math. Probl. Eng..

[35] Herdovics B., Cegla F. (2018). Compensation of phase response changes in ultrasonic transducers caused by temperature variations. Struct. Health Monit..

[36] Guo J., Li B., Liu Z., Hong J., Wu X. (2016). Integration of geometric variation and part deformation into variation propagation of 3-D. assemblies. Int. J. Prod. Res..

[37] Du F., Li B., Hong J. (2015). Application of ultrasound technique to evaluate contact condition on the faying surface of riveted joints. J. Eng. Tribol..

[38] Sevillano E., Sun R., Perera R. (2016). Damage detection based on power dissipation measured with PZT sensors through the combination of electro-mechanical impedances and guided waves. Sensors.

